# The Association Between Subclinical Atherosclerosis Serum Markers and Oxidative DNA Damage in Normoglycemic Normotolerant Offspring of Diabetic Parents

**DOI:** 10.1111/1753-0407.70133

**Published:** 2025-07-30

**Authors:** Masoumeh Rahimi, Farhad Ghadiri Soufi, Shabnaz Koochakkhani, Behnaz Rahnama Inchehsablagh, Abnoos Azarbad, Masoumeh Mahmoudi, Masoumeh Kheirandish, Farideh Jalali Mashayekhi, Ebrahim Eftekhar

**Affiliations:** ^1^ Student Research Committee Faculty of Medicine, Hormozgan University of Medical Sciences Bandar Abbas Iran; ^2^ Endocrinology and Metabolism Research Center Hormozgan University of Medical Sciences Bandar Abbas Iran; ^3^ Molecular Medicine Research Center Hormozgan Health Institute, Hormozgan University of Medical Sciences Bandar Abbas Iran; ^4^ Department of Biochemistry and Genetics Arak University of Medical Sciences Arak Iran

**Keywords:** 8‐OHdG, atherosclerosis, ICAM‐1, offspring of diabetics, ox‐LDL

## Abstract

**Introduction:**

It has been shown that offspring of type 2 diabetic parents have a high risk for developing diabetes and atherosclerosis, but the exact mechanism is unclear. In the present study, the possible association between oxidative stress and subclinical atherosclerosis serum markers in this population was investigated.

**Method:**

LDL/HDL ratio, triglyceride‐glucose index (TyG), atherogenic index of plasma (AIP), single‐point insulin sensitivity estimator (SPISE) index, oxidized LDL (Ox‐LDL), intercellular adhesion molecules (ICAM‐1 and E‐selectin), as well as the marker of oxidative DNA damage were compared among 150 offspring of diabetic parents (90 normoglycemic and normotolerant offspring, 31 offspring with impaired fasting glucose (IFG), and 29 offspring with impaired glucose tolerance (IGT)), and 40 age‐and sex‐matched healthy control individuals. The control subjects were among individuals with no family history of diabetes.

**Results:**

All three groups with diabetic parents, that is, norm‐offspring, IFG, and IGT groups, had higher serum levels of Ox‐LDL, ICAM‐1, and 8‐hydroxy‐2′‐deoxyguanosine (8‐OHdG) than the controls. In the whole population, ICAM‐1 correlated with Ox‐LDL, fasting plasma glucose (FPG) and 8‐OHdG, and Ox‐LDL correlated with LDL/HDL, fasting plasma glucose, TyG index, and 8‐OHdG after adjustment for age, sex, and BMI.

**Conclusion:**

This study shows that subclinical atherosclerosis and oxidative DNA damage are present in normotolerant normoglycemic offspring of type 2 diabetic parents, and they progress with impaired fasting glucose and/or impaired glucose tolerance. Also, our results indicate that a marker of subclinical atherosclerosis, ICAM‐1, was directly correlated with the DNA damage marker, 8‐OHdG.

## Graphical abstract

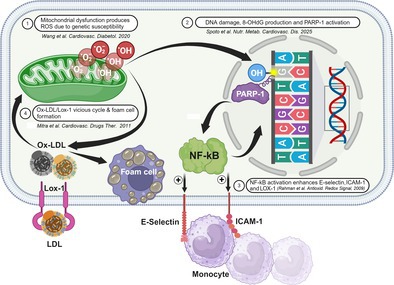

**Summary**

- This study highlights elevated oxidative DNA damage (8‐OHdG) and preclinical atherosclerosis markers (Ox‐LDL, ICAM‐1) in normoglycemic offspring of diabetic parents, indicating increased cardiovascular risk. Early detection of these biomarkers can guide preventive strategies to mitigate diabetes‐related complications.

## 1 Introduction

Cardiovascular disease (CVD) is the leading cause of morbidity and mortality in individuals with diabetes, with a two‐ to four‐fold increase in CVD risk due to extensive vascular complications [1]. Studies indicate that atherosclerosis often begins before type 2 diabetes mellitus (T2DM) is clinically diagnosed, suggesting that vascular damage is already underway in the prediabetic stage [2]. As a chronic inflammatory process, atherosclerosis progresses silently over time, often remaining undetected for years before manifesting as symptomatic disease, a stage referred to as subclinical atherosclerosis [2, 3]. Several risk factors, such as dyslipidemia, hypertension, hyperglycemia, and obesity, contribute not only to endothelial dysfunction and vascular inflammation, but also to insulin resistance [4]. Notably, the mechanisms driving insulin resistance leading to T2DM resemble those underlying endothelial dysfunction and atherosclerosis [5].

Oxidative stress plays a main role in the initiation of diabetes, impaired fasting glucose (IFG), and impaired glucose tolerance (IGT) [6, 7]. Increased oxidative damage in T2DM is linked to lipid peroxidation, excessive reactive oxygen species (ROS) production, and DNA oxidation, which contribute to vascular dysfunction and atherogenesis [8]. DNA oxidation, in turn, in telomeric or non‐telomeric DNA regions, can accelerate cellular aging and lead to endothelial dysfunction and atherosclerosis [9]. T2DM results from complex interactions between environmental and genetic factors, and its prevalence is increasing rapidly due to lifestyle changes [10].

Genetic predisposition plays a critical role, as first‐degree relatives of individuals with T2DM have a 40% lifetime risk of developing the disease, which rises to nearly 70% if both parents are affected [11]. Moreover, those with a family history of diabetes (FHD) have a significantly higher likelihood of developing prediabetes [12]. Given their shared genetic background with T2DM patients, normoglycemic individuals with FHD represent an ideal population for studying early metabolic disturbances before hyperglycemia develops [13]. The Multi‐Ethnic Study of Atherosclerosis (MESA) identifies FHD as an independent cardiovascular risk factor [14], and research indicates that first‐degree relatives of diabetic patients are more susceptible to endothelial dysfunction and early atherosclerosis [1].

The objectives of the present study were: (1) evaluating oxidative stress markers (8‐hydroxy‐2′‐deoxyguanosine; 8‐OHdG and oxidized LDL; Ox‐LDL) and intercellular adhesion molecules (ICAM‐1 and E‐selectin) as early endothelial dysfunction markers in normoglycemic offspring of patients with T2DM, (2) investigating the possible relationship between these biomarkers, and (3) comparing these markers across different glucose tolerance statuses (IFG and IGT).

The selection of these biomarkers is based on the fact that, among the various oxidative stress and antioxidant markers studied (e.g., malondialdehyde (MDA), 8‐isoprostane, protein carbonyl, glutathione, superoxide dismutase, etc.), 8‐hydroxy‐2′‐deoxyguanosine (8‐OHdG) stands out due to its higher specificity for oxidative DNA damage, making it a more reliable indicator of oxidative stress [15], and showing a strong correlation with subclinical atherosclerosis progression and vascular inflammation [16]. Similarly, Ox‐LDL provides unique insights into both oxidative damage and vascular risk, showing particular predictive value in prediabetic populations [17]. ICAM‐1 serves as our primary endothelial activation marker due to its: (i) early response to oxidative stress [18], (ii) consistent multi‐ethnic association with plaque progression [19], and (iii) therapeutic modifiability [20]. E‐selectin completes this panel as it is an endothelial adhesion molecule specifically expressed on activated endothelial cells in response to inflammatory stimuli, serving as a sensitive biomarker of early endothelial activation—a key initiating event in vascular inflammation and atherogenesis [21].

Collectively, these biomarkers capture the pathological continuum from nuclear DNA damage (8‐OHdG) and lipid peroxidation (Ox‐LDL) to endothelial dysfunction (ICAM‐1/E‐selectin), providing a comprehensive insight into preclinical vascular pathology in high‐risk populations.

While previous studies have examined individual biomarkers of oxidative stress or endothelial dysfunction, this is the first study to simultaneously assess the triad of nuclear DNA damage, oxidized lipids, and endothelial activation markers in normoglycemic and prediabetic offspring of T2DM patients.

## 2 Materials and Methods

150 non‐diabetic offspring of mothers or fathers with type 2 diabetes (mean age 31.4 ± 4.2 years; 60 men and 90 women; BMI 26.1 ± 2.4 kg/m^2^) and 40 normoglycemic normotolerant control individuals with no family history of diabetes (mean age 29.49 ± 5.84 years; 19 men and 21 women; BMI 23.80 ± 2.96 kg/m^2^) were recruited for this study. Sample size was calculated using GPower 3.1 (α = 0.05, power = 80) based on Ox‐LDL differences (d = 0.5) from a prior study [22]. The minimum required sample was 120 participants (30 per group); we enrolled 150 to account for attrition.

The offspring of diabetic parents were defined as individuals whose parents were diagnosed with type 2 diabetes mellitus for at least 1 year and were on antidiabetic treatment. This was verified through a questionnaire and interview. The offspring of diabetic parents were recruited from patients admitted to the outpatient clinic of Endocrinology and Metabolism at Shahid Mohammadi Hospital, Bandar Abbas, and the control group was selected from the hospital staff. None of the participants was related to each other. Subjects with any known disease, infection, dyslipidemia, pregnancy, lactation, use of medications, or antioxidant supplements, alcohol consumption, or smoking within the last 3 months were excluded.

Participants were divided into four groups: normoglycemic normotolerant offspring of diabetic parents (norm‐offspring, *n* = 90), prediabetic offspring of diabetic parents with impaired fasting glucose (IFG, *n* = 31), prediabetic offspring of diabetic parents with impaired glucose tolerance test (IGT, *n* = 29), and healthy control people with no family history of diabetes (control, *n* = 40).

After an overnight fast, all participants had undergone a standard 2‐h, 75‐g oral glucose tolerance test (OGTT). Prediabetes was defined if the fasting plasma glucose (FPG) level was 100–125 mg/dL (defined as IFG) or the glucose level 2 h after an OGTT was 140–199 mg/dL (defined as IGT).

The subject's body weights were assessed using calibrated electronic weighing scales (Tanita TBF 300, TANITA Company, Japan), and their heights were measured by a portable stadiometer, and then BMI was calculated.

Overnight fasting blood samples (6 mL) were taken, and the serum was isolated and stored at −80°C for biochemical measurements. Glucose and lipid profiles were assessed on the chemistry autoanalyzer BT1500 (Biotechnical Instruments, Rome, Italy) using a standard kit (Para Azmoon company, Iran).

For the calculation of the atherogenic index of plasma (AIP), the log_10_ (TG/HDL) formula was used. The triglyceride‐glucose (TyG) index, a marker of insulin resistance, was calculated as a formula: ln [fasting triglycerides (mg/dL) × fasting plasma glucose (mg/dL)/2]. The single‐point insulin sensitivity estimator (SPISE) index was calculated as: 600 × HDL^0.185^/(triglycerides^0.2^ × BMI^1.338^).

Serum concentration of ICAM‐1, E‐Selectin, Ox‐LDL, and 8‐OHdG was measured using the ELISA method and standard kits (Wuhan EIabScience company, China). For Ox‐LDL, ICAM‐1, and 8‐OHdG kits, the intra‐assay and inter‐assay coefficients of variation (CV) were < 7% and < 8%, and for E‐selectin, the intra‐assay and inter‐assay CV were < 6% and < 8%, respectively. The sensitivity for Ox‐LDL, ICAM‐1, E‐selectin, and 8‐OHdG kits was 37 pg/mL, 0.19 ng/mL, 46.88 pg/mL, and 0.94 ng/mL, respectively. All measurements were performed according to the manufacturers' protocols, using an ELISA reader (Anthos 2020, Biochrome France) at 450 nm.

The ethics committee of the Hormozgan University of Medical Sciences (code No: IR.HUMS.REC.1398.474) confirmed the study protocol, and all subjects gave written informed consent.

## 3 Statistical Analysis

Data were analyzed using IBM SPSS Statistics software (version 26.0, USA). The Shapiro–Wilk test was conducted to evaluate the normality of the data distribution. Data are presented as means ± standard deviation (SD). The analysis of variance (ANOVA) test was used to compare normally distributed variables. Between‐group analyses for non‐normally distributed variables (Ox‐LDL and TyG index) were performed using both the Kruskal–Wallis test (for means) and the independent‐samples median test (for medians). They were considered significant only when between‐group differences for both mean and median were significant. The Tukey post hoc test was used to determine which group was the cause of the difference. The linear regression test was done to find the correlation between variables. All analyses were two‐tailed, and *p*‐values less than 0.05 were considered significant.

## 4 Results

### 4.1 Clinical Features of the Study Participants

The clinical features of the study participants are presented in Table 1. No significant differences in sex, age, systolic, and diastolic blood pressure were detected among the study groups. Prediabetic individuals in both the IFG and IGT groups had higher BMI than the controls (*p* = 0.001 and *p* = 0.002, respectively). Also, the individuals with IFG had a higher mean BMI compared with norm‐offspring individuals (*p* = 0.037), but this variable was not statistically significant between norm‐offspring and control groups (*p* = 0.23).

**TABLE 1:** Characteristics of four groups participated in this study.

Variable	Control (*n* = 40)	Norm‐offspring (*n* = 89)	IFG offspring (*n* = 31)	IGT offespring (*n* = 29)
Sex (male/female)	19/21	30/59	15/16	14/15
Age (years)	29.49 ± 5.84	30.15 ± 7.61	34.80 ± 4.88	31.39 ± 8.16
BMI (Kg/m^2^)	23.80 ± 2.96	25.34 ± 4.05	27.52 ± 3.33*^,##^	27.68 ± 3.70*
FPG (mg/dl)	89.57 ± 6.71	87.38 ± 7.40	106.30 ± 5.77*^,#,$^	99.33 ± 3.56*^,#^
2‐h OGTT (mg/dL)	110.09 ± 20.93	118.56 ± 21.44	115.12 ± 24.17	168.83 ± 21.19**^,##,&^
Systolic blood pressure (mmHg)	108.12 ± 9.14	107.38 ± 10.01	111.12 ± 7.13	109.71 ± 9.11
Diastolic blood pressure (mmHg)	68.11 ± 8.96	69.17 ± 9.81	70.07 ± 8.91	69.44 ± 10.21

*Note:* Data are presented as means ± SD. *: *p* < 0.01 versus control group; **: *p* < 0.05 versus control group; #: *p* < 0.01 versus norm‐offspring group; ##: *p* < 0.05 versus norm‐offspring group; $: *p* < 0.05 versus IGT group; &: *p* < 0.05 versus IFG group.

Abbreviations: IFG, offspring with impaired fasting glucose; IGT, offspring with impaired glucose tolerance test; Norm‐offspring, normoglycemic normotolerant offspring.

Prediabetic offspring with IFG had higher FPG than control, norm‐offspring, and IGT groups (*p* = 0.0001, *p* = 0.0001, and *p* = 0.021, respectively), and the prediabetic offspring with IGT had higher 2‐h OGTT level than control, norm‐offspring, and IFG groups (*p* = 0.001, *p* = 0.007, and *p* = 0.003, respectively). Fasting plasma glucose and 2‐h OGTT level were not significantly different between norm‐offspring and control groups (*p* = 0.42).

### 4.2 Preclinical Atherosclerosis Serum Markers

Table 2 shows the mean ± SD values of the preclinical atherosclerosis serum markers (LDL/HDL ratio, Ox‐LDL, ICAM‐1, E‐selectin, API, and TyG and SPISE indices) in the study groups. While the norm‐offspring group had a higher level of Ox‐LDL and ICAM‐1 than the controls (*p* = 0.012 and *p* = 0.039, respectively), the LDL/HDL ratio, E‐selectin, API, TyG, and SPISE indices were not statistically different between norm‐offspring and control groups.

**TABLE 2:** Comparison of preclinical atherosclerosis serum markers among norm‐offspring, prediabetic, and control groups.

Variable	Control (*n* = 40)	Norm‐offspring (*n* = 89)	IFG (*n* = 31)	IGT (*n* = 29)
LDL/HDL ratio	1.76 ± 0.41	1.86 ± 0.53	2.19 ± 0.52*^,##^	2.03 ± 0.47
AIP	0.28 ± 0.28	0.27 ± 0.23	0.44 ± 0.27^##^	0.35 ± 0.28
SPISE index	5.69 ± 1.51	5.78 ± 1.01	6.95 ± 1.76	7.42 ± 1.84*
TyG index	8.33 ± 0.53	8.33 ± 0.48	8.87 ± 0.56*^,#^	8.67 ± 0.69
Ox‐LDL (ng/mL)	32.20 ± 19.28	44.38 ± 15.45**	61.12 ± 27.64*^,#^	79.61 ± 35.64*^,#,&^
ICAM‐1 (ng/mL)	60.64 ± 29.81	96.49 ± 46.72**	110.33 ± 42.80*	125.30 ± 48.78*^,##^
E‐Selectin (ng/mL)	41.42 ± 22.01	49.02 ± 22.95	53.91 ± 28.79	63.50 ± 34.95

*Note:* Data are presented as means ± SD. *: *p* < 0.01 versus control group; **: *p* < 0.05 versus control group; #: *p* < 0.01 versus norm‐offspring group; ##: *p* < 0.05 versus norm‐offspring group; &: *p* < 0.05 versus IFG group.

Abbreviations: AIP, atherogenic index of plasma; IFG, offspring with impaired fasting glucose; IGT, offspring with impaired glucose tolerance test; Norm‐offspring, normoglycemic normotolerant offspring; SPISE, single‐point insulin sensitivity estimator index; TyG, triglyceride‐glucose index.

Both IFG and IGT groups had significantly higher Ox‐LDL and ICAM‐1 levels than the control group (*p* < 0.01 for all comparisons). The IFG group had significantly higher LDL/HDL ratio, Ox‐LDL, API, and TyG index compared to the norm‐offspring group (*p* = 0.017, *p* = 0.006, *p* = 0.013, and *p* = 0.001, respectively). On the other hand, the IGT group had significantly higher levels of Ox‐LDL, ICAM‐1, and TyG index than the norm‐offspring group (*p* = 0.004, *p* = 0.011, and *p* = 0.028, respectively). Also, the SPISE index shows significantly higher values in the IGT group than controls (*p* = 0.008). Finally, a higher serum Ox‐LDL level was noted in the IGT group compared to the IFG group (*p* = 0.042).

### 4.3 Serum Biomarker of Oxidative DNA Damage

**FIGURE 1:**
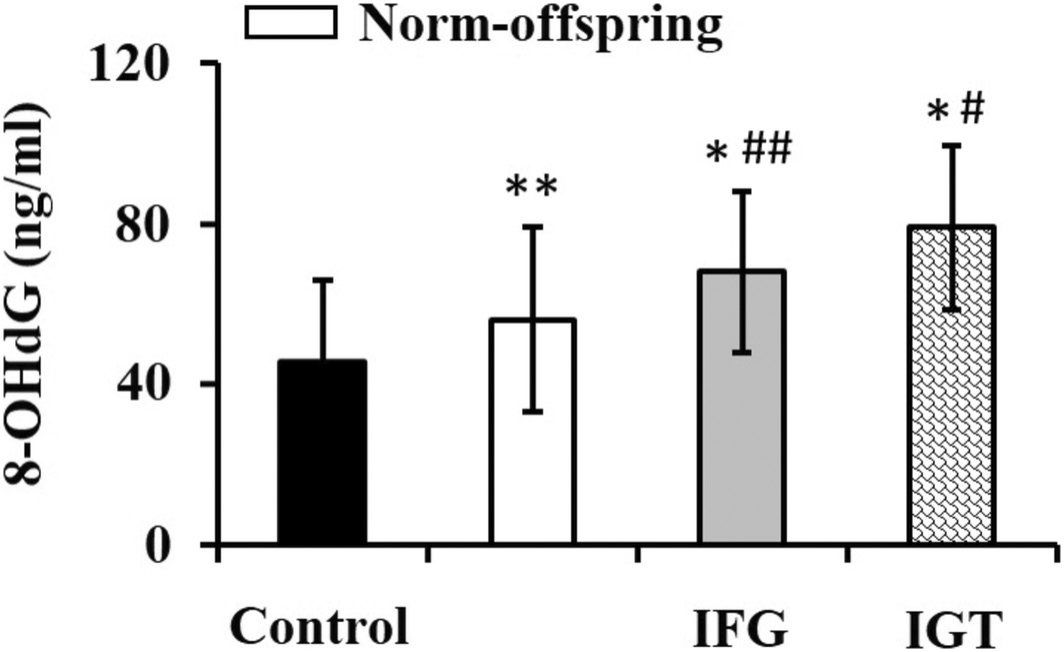
The serum concentration of 8‐OHdG as the marker of oxidative DNA damage in the study groups. Data are presented as means ± SD. Abbreviations and symbols: Norm‐offspring: Normoglycemic normotolerant offspring; IFG: Offspring with IFG; IGT: Offspring with IGT. *: *P* < 0.01 versus control group; **: *P* < 0.05 versus control group; #: *P* < 0.01 versus norm‐offspring group; ##: *P* < 0.05 versus norm‐offspring group.

The result of serum concentration of 8‐OHdG as a marker of oxidative DNA damage is presented in Figure 1. All three groups with diabetic parents, that is, norm‐offspring, IFG, and IGT groups, had higher 8‐OHdG serum concentrations than the control subjects (*p* = 0.041, *p* = 0.003 and *p* = 0.001, respectively). Moreover, both IFG and IGT groups had significantly higher 8‐OHdG levels than the norm‐offspring group (*p* = 0.019 and *p* = 0.003, respectively).

### 4.4 Correlations Between Preclinical Atherosclerosis Serum Markers and 8‐OHdG

Linear regression analysis showed that ICAM‐1 correlated with Ox‐LDL (*p* = 0.009) and 8‐OHdG (*p* = 0.002) in the norm‐offspring group, after adjustment for age, sex, and BMI (Table 3), whereas in the prediabetic offspring groups, only a correlation between ICAM‐1 and LDL/HDL ratio was observed in the IGT group (*p* = 0.018).

**TABLE 3:** Linear regression analysis of Ox‐LDL and ICAM‐1 after adjusting for age, sex and BMI.

Independent variable	Norm‐offspring (*n* = 89)		IFG (*n* = 31)		IGT (*n* = 29)	
	*β* (SE)	*p*	*β* (SE)	*p*	*β* (SE)	*p*
Dependent variable: Ox‐LDL						
LDL\HDL	0.09 (0.02)	0.08	0.13 (0.17)	0.45	0.09 (0.08)	0.09
8‐OHdG	0.16 (0.12)	0.13	0.31 (0.17)	0.08	0.19 (0.10)	0.07
FPG	0.10 (0.11)	0.39	0.36 (0.24)	0.32	0.15 (0.08)	0.10
AIP	0.13 (0.11)	0.30	0.06 (0.10)	0.59	0.23 (0.14)	0.11
SPISE	−0.14 (0.12)	0.26	−0.14 (0.12)	0.22	−0.20 (0.14)	0.17
TyG index	0.10 (0.11)	0.41	0.31 (0.22)	0.17	0.18 (0.12)	0.09
Dependent variable: ICAM‐1						
LDL\HDL	0.34 (0.17)	0.05	0.51 (0.39)	0.10	0.55 (0.22)	0.01
8‐OHdG	0.46 (0.17)	0.002	0.31 (0.17)	0.08	0.31 (0.23)	0.46
FPG	0.17 (0.13)	0.09	0.28 (0.15)	0.13	0.20 (0.15)	0.29
AIP	0.23 (0.15)	0.14	0.17 (0.15)	0.14	0.40 (0.27)	0.14
SPISE	−0.38 (0.25)	0.07	−0.26 (0.16)	0.07	−0.32 (0.20)	0.07
TyG index	0.17 (0.15)	0.31	0.11 (0.07)	0.31	0.21 (0.15)	0.20
Ox‐LDL	0.48 (0.15)	0.009	0.30 (0.18)	0.05	0.26 (0.16)	0.08

*Note:* Abbreviations and symbols are like Table 2.

**FIGURE 2:**
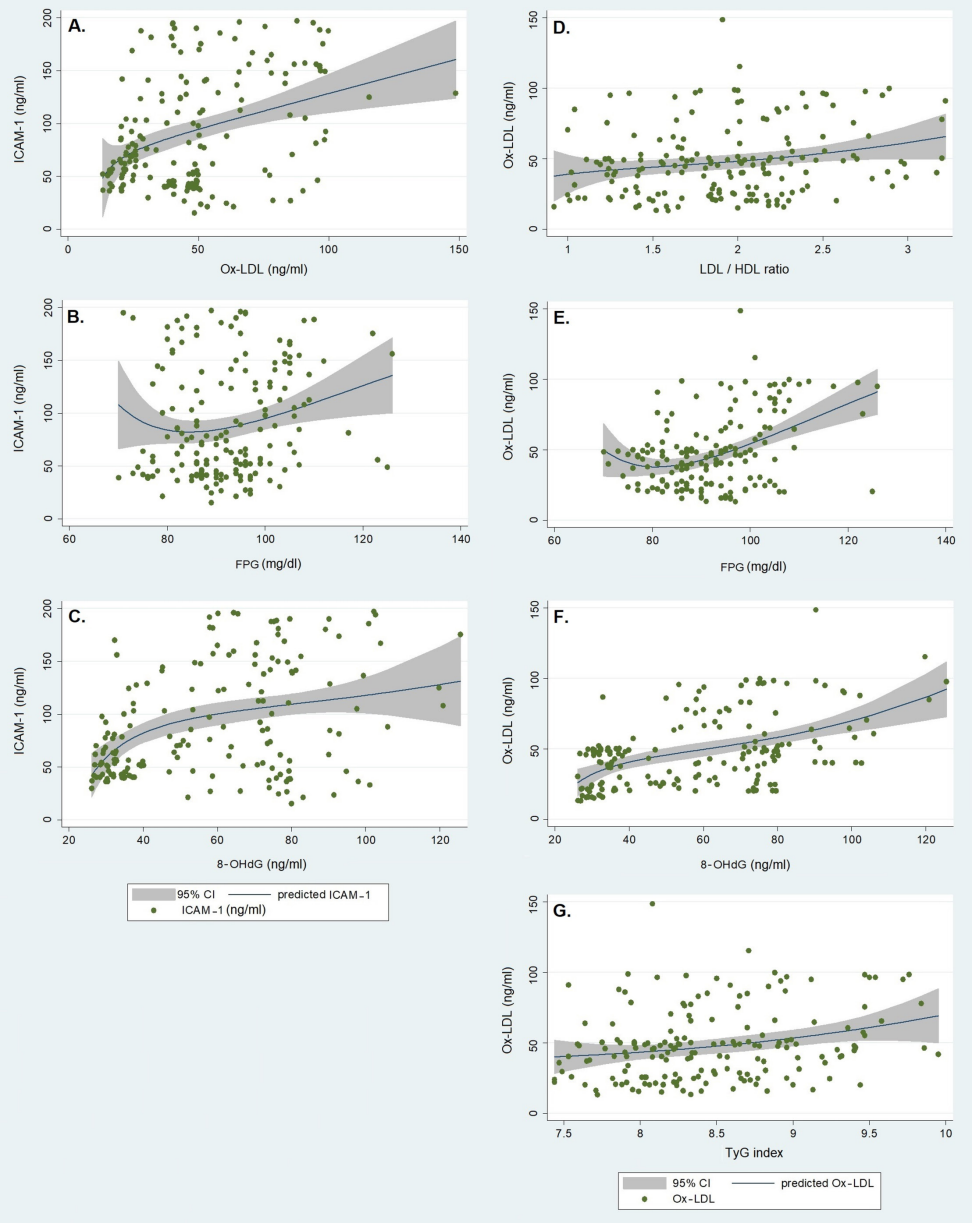
The linear regression analysis in all offspring of diabetic parents participated in this study (*n* = 149) showing ICAM‐1 positively correlated with (A) Ox‐LDL (*p* = 0.003), (B) fasting plasma glucose, FPG (*p* = 0.025) and (C) 8‐OHdG (*p* = 0.002), and also Ox‐LDL positively correlated with (D) LDL/HDL ratio (*p* = 0.009), (E) FPG (*p* = 0.005), (F) 8‐OHdG (*p* = 0.001) and (G) TyG index (*p* = 0.002). Abbreviations and symbols are like Table 2.

Also, in the whole population, linear regression analysis showed that ICAM‐1 correlated with Ox‐LDL (*p* = 0.003), FPG (*p* = 0.025), and 8‐OHdG (*p* = 0.002), and Ox‐LDL correlated with LDL/HDL ratio (*p* = 0.009), FPG (*p* = 0.005), TyG index (*p* = 0.002) and 8‐OHdG (*p* = 0.001) after adjustment for age, sex, and BMI (Figure 2).

## 5 Discussion

Coronary heart disease and diabetes mellitus are major related public health concerns in which genetic susceptibility plays an important role [23]. It would be noteworthy to investigate whether early impairments in metabolic status and vascular function can be recognized in the normoglycemic offspring of diabetic patients who may be at higher risk than the general population for serious health complications. Hence, in the present investigation, we evaluated the risk of atherosclerosis in the normoglycemic normotolerant offspring of diabetic patients (norm‐offspring) by measuring oxidative DNA damage marker (8‐OHdG) and early preclinical atherosclerosis serum markers, including Ox‐LDL and intercellular adhesion molecules (ICAM‐1 and E‐selectin) and comparing them with data of prediabetic and control groups. Our goal was to offer a new insight into the initiation and progression of atherosclerosis before the onset of diabetes and to raise awareness for preventing disease progression and its related complications.

Our study reveals a progressive continuum of metabolic and vascular dysfunction in offspring of diabetic parents, beginning with oxidative stress in normoglycemic individuals and advancing to endothelial injury in prediabetic states. Elevated 8‐OHdG, Ox‐LDL, and ICAM‐1 levels in normoglycemic offspring suggest that these metabolic alterations emerge well before overt hyperglycemia. These findings support the concept of “diabetic vasculopathy”, where molecular abnormalities precede clinical manifestations of diabetes [24]. The graded metabolic disturbances observed align with the hypothesis that a family history of diabetes predisposes individuals to early vascular and metabolic changes, increasing their cardiometabolic risk.

Regarding anthropometric measurements, both IFG and IGT groups had significantly higher BMI than controls (*p* = 0.001 and *p* = 0.002, respectively), while normoglycemic offspring showed a non‐significant trend (*p* = 0.23). This aligns with Meigs et al. [25], who reported increased adiposity in offspring of diabetic parents before glucose dysregulation. The elevated BMI in prediabetic groups likely reflects both genetic predisposition and shared familial lifestyle factors contributing to positive energy balance.

Our findings show that even normoglycemic offspring exhibited significantly higher 8‐OHdG levels than controls (*p* = 0.014), with the highest elevation in the IGT group (*p* = 0.001). These findings are in agreement with a previous study showing that normoglycemic offspring of diabetic patients exhibit higher 8‐OHdG levels even in the absence of hyperglycemia [26]. This observation confirms that genetically predisposed individuals are at higher risk of oxidative DNA damage. The particularly high 8‐OHdG levels in IGT subjects may reflect greater postprandial oxidative stress, consistent with the “glucose toxicity” paradigm [27].

Ox‐LDL plays a vital role in the development of atherosclerosis by promoting the infiltration and migration of monocytes, as well as the proliferation of smooth muscle cells [28]. The results of this study reveal that Ox‐LDL and ICAM‐1 levels were elevated in all offspring groups, with a progressive increase from normoglycemic to prediabetic individuals. However, the levels of E‐selectin were not markedly different among the three studied groups. This pattern supports the “common soil” hypothesis [29], suggesting shared genetic and environmental factors underlying diabetes and cardiovascular disease.

Our results confirm previously published findings, where higher levels of circulating Ox‐LDL have been observed in prediabetic patients compared to normoglycemic control subjects [30, 31]. Moreover, circulating Ox‐LDL is a marker of cardiovascular disease [32]. Behzadi et al. [22] reported a markedly higher concentration of Ox‐LDL in diabetic subjects than in controls, confirming oxidative stress in diabetes. They also observed elevated Ox‐LDL levels in the first‐degree relatives of diabetic patients compared to the controls, which is consistent with our results. However, they found no significant differences in Ox‐LDL levels between the normoalbuminuric diabetic subjects and the group of first‐degree relatives. According to their study, genetic predisposition and oxidative stress influence LDL oxidation.

Although elevated levels of ICAM‐1 and E‐selectin are generally associated with increased cardiovascular risk, studies in people with a familial predisposition to T2DM have yielded inconsistent findings. Our results are consistent with those of McSorley et al. [33], who reported significantly higher ICAM‐1 levels in normoglycemic offspring of diabetic parents, with no significant difference in E‐selectin levels compared to controls. Bannan et al. [34] observed elevated E‐selectin concentration in first‐degree relatives of T2DM patients—a finding that stands in contrast to our results. This inconsistency may be attributed to the complex interaction between genetic background, environmental influences, and disease progression, emphasizing the importance of comprehensive investigation to determine the reliability of these findings.

**FIGURE 3:**
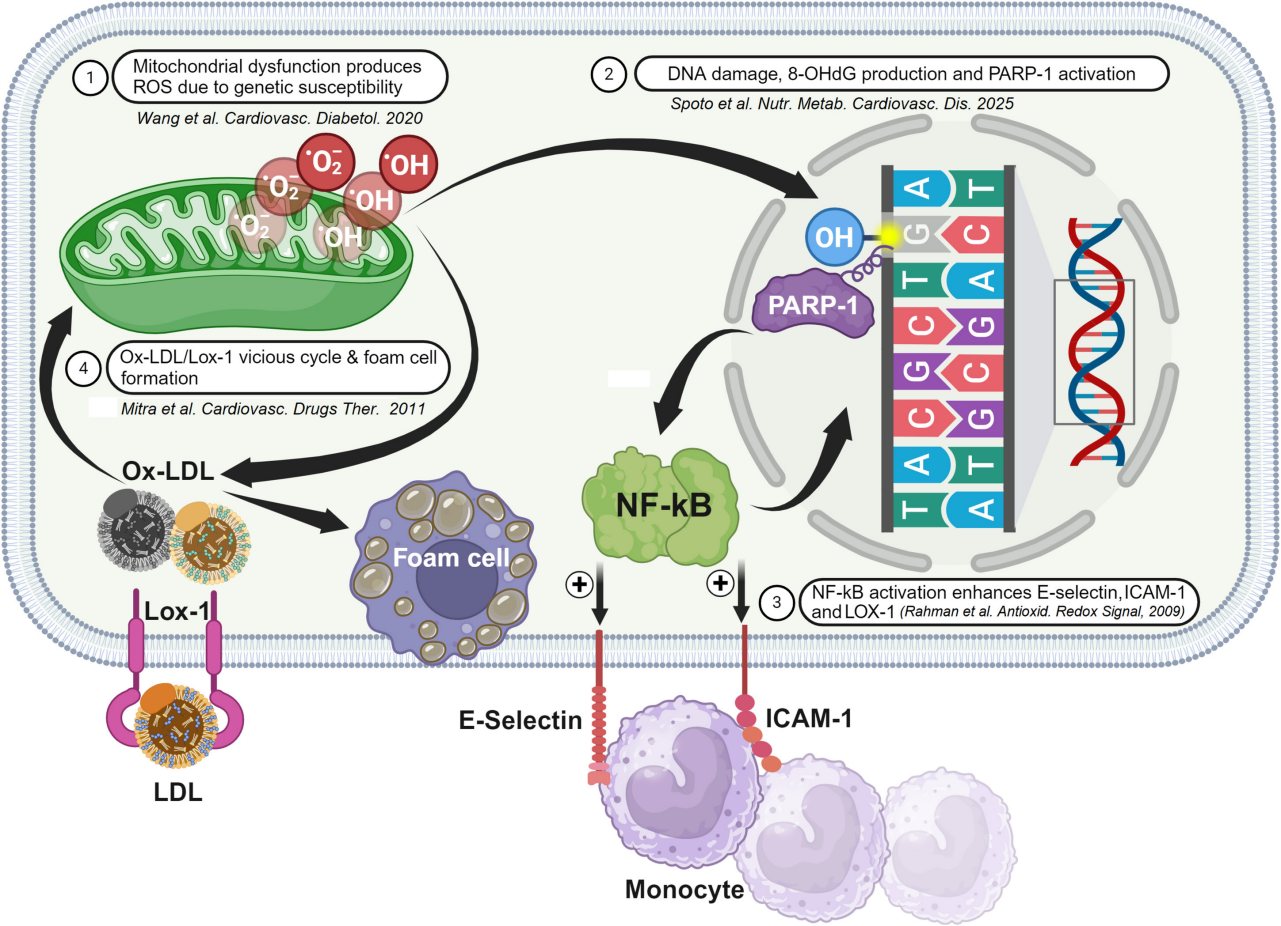
Proposed molecular mechanism linking reactive oxygen species‐driven mitochondrial dysfunction to atherosclerosis in offspring of diabetic individuals. (1) Mitochondrial dysfunction (due to genetic susceptibility and hyperglycemia) overproduces reactive oxygen species (ROS). (2) ROS induce DNA damage (elevated 8‐OHdG) and activate PARP‐1, depleting NAD+ and impairing endothelial function. (3) NF‐KB activation upregulates adhesion molecules (E‐selectin, ICAM‐1) and the LOX‐1 receptor, amplifying vascular inflammation. (4) The Ox‐LDL/LOX‐1 vicious cycle promotes foam cell formation and atherosclerotic plaque progression.

There is no clear mechanistic explanation for the endothelial dysfunction that was seen in first‐degree relatives of diabetic patients. The condition is multifactorial, involving a mix of genetic influences and different components of metabolic syndrome, such as insulin resistance, oxidative stress, dyslipidemia, and hypertension [35]. The generation of ROS and the resulting oxidative stress are crucial in the development of atherosclerotic cardiovascular diseases. It is well established that cells exposed to ROS show frequent DNA damage [36]. The observed increase in 8‐OHdG levels may be indicative of mitochondrial dysfunction and impaired DNA repair (Figure 3, Stage 1), consistent with the findings of Wang et al. study [37]. 8‐OHdG promotes telomere shortening and mitochondrial dysfunction via PARP‐1 activation, while oxidative damage triggers cellular senescence [38] and vascular inflammation through NF‐κB‐mediated ICAM‐1 upregulation [39] (Figure 3, Stages 2 and 3). Additionally, the observed Ox‐LDL/ICAM‐1 correlation supports the LOX‐1/NF‐κB pathway [40], which perpetuates oxidative stress through a feed‐forward ROS generation loop [41] (Figure 3, Stage 4). Moreover, the significant correlation between Ox‐LDL and LDL/HDL ratio (*p* = 0.009) reinforces the role of atherogenic dyslipidemia in early vascular damage.

Another finding of the present study is that in the norm‐offspring group, there was a significant positive association between 8‐OHdG and Ox‐LDL with ICAM‐1, while this association was not present in the prediabetic subjects (Table 3). Norm‐offspring of diabetic parents may exhibit early signs of metabolic dysregulation due to genetic or environmental factors. In this group, oxidative stress may be more pronounced, leading to a significant correlation with inflammation (ICAM‐1). Conversely, individuals with prediabetes may already have altered metabolic pathways that diminish the relationship between oxidative stress and inflammation [42]. In addition, norm‐offspring might still have intact compensatory mechanisms that respond to oxidative stress, leading to an observable relationship with inflammation markers. In contrast, subjects with prediabetes might have already activated chronic inflammatory pathways that obscure the correlation with oxidative stress markers [43].

The TyG index, an insulin resistance marker, was significantly elevated in IFG subjects compared to normoglycemic offspring, while the SPISE index was higher in the IGT group (*p* = 0.008 vs. controls). These different associations suggest that various insulin resistance indices may capture distinct metabolic dysfunction patterns in different prediabetic phenotypes, as noted by Barbu et al. [44].

Our findings may have important clinical implications. (1) The presence of vascular risk markers in normoglycemic offspring suggests that cardiovascular risk assessment should begin early, even before glucose abnormalities appear. (2) Oxidative stress and vascular inflammation markers should be included in cardiovascular risk evaluations, rather than relying solely on traditional factors. (3) The distinct metabolic profiles of IFG and IGT highlight the need for tailored prevention strategies for these prediabetic subgroups. (4) The significant role of oxidative stress underscores the potential benefits of early interventions, such as lifestyle modifications or targeted antioxidant therapies, to delay or prevent metabolic and vascular complications in this high‐risk population.

Despite its strengths, our study has some limitations. It was conducted in a homogeneous Iranian population, which may limit its generalizability. Ethnic variations may significantly influence the relationship between oxidative biomarkers and cardiovascular risk [19]. Future multicenter studies across diverse populations would help validate our findings and establish universal cutoff values for these biomarkers in individuals at risk for diabetes. Additionally, the cross‐sectional design of the study prevents establishing causality between oxidative stress and cardiovascular risk. The relatively small sample size of the normoglycemic offspring group and the absence of advanced vascular imaging (e.g., carotid IMT) or detailed family history data further limit comprehensive risk assessment. Future longitudinal studies with repeated oxidative stress and vascular function assessments will be essential to better understand the progression of these early abnormalities.

## 6 Conclusion

To the best of our knowledge, this is the first investigation in which the serum level of both 8‐OHdG and ICAM‐1 was studied in normoglycemic normotolerant offspring of diabetic patients, prediabetic, and control subjects. Our findings clearly show that ICAM‐1, as a marker of subclinical atherosclerosis, was directly correlated with 8‐OHdG as a DNA damage marker. In addition, our results show that normoglycemic normotolerant offspring of diabetic patients are associated with oxidative DNA damage, which puts these subjects at an increased risk of developing atherosclerosis. Our study demonstrates that normoglycemic offspring of diabetic parents exhibit distinct vascular and oxidative stress abnormalities despite maintaining normal glucose regulation. These findings suggest that cardiovascular risk develops early in diabetes‐prone individuals, emphasizing the need for targeted screening and prevention strategies well before glucose abnormalities emerge.

## Author Contributions

B.R.I. and S.K. performed experiments; M.M., M.R., and A.A. analyzed the data; E.E., F.G.S., M.K., and F.J.M. interpreted the results of experiments and designed the protocol; F.G.S. and E.E. wrote the paper, and all authors approved the final draft of the manuscript.

## Conflicts of Interest

The authors declare there is no conflicts of interest.

## Data Availability

Data will be made available on reasonable request.
